# PPAR-γ Modulators as Current and Potential Cancer Treatments

**DOI:** 10.3389/fonc.2021.737776

**Published:** 2021-09-23

**Authors:** Tiange Chi, Mina Wang, Xu Wang, Ke Yang, Feiyu Xie, Zehuan Liao, Peng Wei

**Affiliations:** ^1^ School of Traditional Chinese Medicine, Beijing University of Chinese Medicine, Beijing, China; ^2^ First Clinical Medical School, Beijing University of Chinese Medicine, Beijing, China; ^3^ Department of Acupuncture and Moxibustion, Beijing Key Laboratory of Acupuncture Neuromodulation, Beijing Hospital of Traditional Chinese Medicine, Capital Medical University, Beijing, China; ^4^ Oncology Department, Wangjing Hospital of China Academy of Chinese Medical Sciences, Beijing, China; ^5^ School of Biological Sciences, Nanyang Technological University, Singapore, Singapore; ^6^ Department of Microbiology, Tumor and Cell Biology (MTC), Karolinska Institutet, Stockholm, Sweden

**Keywords:** PPAR-γ, PPAR-γ modulators, PPAR-γ agonists, PPAR-γ antagonists, cancer treatment

## Abstract

Worldwide, cancer has become one of the leading causes of mortality. Peroxisome Proliferator-Activated Receptors (PPARs) is a family of critical sensors of lipids as well as regulators of diverse metabolic pathways. They are also equipped with the capability to promote eNOS activation, regulate immunity and inflammation response. Aside from the established properties, emerging discoveries are also made in PPAR’s functions in the cancer field. All considerations are given, there exists great potential in PPAR modulators which may hold in the management of cancers. In particular, PPAR-γ, the most expressed subtype in adipose tissues with two isoforms of different tissue distribution, has been proven to be able to inhibit cell proliferation, induce cell cycle termination and apoptosis of multiple cancer cells, promote intercellular adhesion, and cripple the inflamed state of tumor microenvironment, both on transcriptional and protein level. However, despite the multi-functionalities, the safety of PPAR-γ modulators is still of clinical concern in terms of dosage, drug interactions, cancer types and stages, etc. This review aims to consolidate the functions of PPAR-γ, the current and potential applications of PPAR-γ modulators, and the challenges in applying PPAR-γ modulators to cancer treatment, in both laboratory and clinical settings. We sincerely hope to provide a comprehensive perspective on the prospect of PPAR-γ applicability in the field of cancer treatment.

## 1 Introduction

Cancer, among all health issues, is a leading concern worldwide. According to the American Cancer Society, by 2021, it is the second most common cause of death in the US, exceeded only by heart disease. It is estimated that in 2021, approximately 1.9 million new cases and over 608,000 deaths of cancer are expected in the U.S. (1). It is believed that by 2025, more than 20 million new cancer cases are expected to occur annually (2). The well-established risk factors include genetic susceptibility, ionizing radiation, infections, smoking, insobriety, inappropriate diet, sedentary lifestyle, obesity, and other unclassified carcinogenic environmental exposures, which have been promoting cancer prevalence (3). There are disparities in the morbidity and mortality of cancer among populations of different socio-economic backgrounds: in high-income countries, that of various cancers have been declining over the past decade, owing a lot to efficient screening, early detection, and more effective treatments; however, they increased in low- and middle-income countries, resulting from increasing rate of smoking, inappropriate diet, lack of physical activity and infections (4). Compared to the most developed countries, death rates of cervical cancer in the most underdeveloped countries were 2-fold higher, and 40% higher for men with lung and liver cancers from 2012 to 2016 (5).

Peroxisome Proliferator-Activated Receptors (PPARs) are ligand-inducible transcription factors, belonging to the nuclear receptor superfamily (6, 7). Upon ligand-binding, PPARs translocate to the nucleus, after binding to peroxisome proliferator response elements (PPREs) on DNA and heterodimerizing with retinoid X receptor, they modulate the transcription of target genes. PPARs have long established properties in lipid and glucose metabolism and homeostasis regulation. In the meantime, evidence is mounting in their strong candidacy as modulators in fields including immunity and inflammation, vascular functions, cellular proliferation, differentiation, development, and apoptosis (8). In mammals, PPARs has three subtypes: PPAR-α, PPAR-γ and PPAR-β/δ. Three subtypes of PPARs are highly identical in biological structure. Nevertheless, their biological functions, tissue distributions and ligand affinities show considerable distinctions (9). PPAR-γ, with two isoforms of PPARγ1 and PPARγ2, is most highly expressed in adipose tissues, regulating glucose and lipid homeostasis, insulin sensitivity, adipogenesis, inflammation, immune response, and tumorigenesis. Of those said isoforms, PPARγ1 is dominant and more universally expressed in various tissues upon biological activation, while PPARγ2 is primarily restricted to adipose tissue physiologically, but can be induced in other tissues by high-fat diet (HFD), derived from four different mRNA species (PPARG1, PPARG2, PPARG3 and PPARG4) (10, 11).

Cancers are characterized by aberrated gene mutations, abnormal cell metabolism, low differentiation rate and exceptionally high growth rate (12–14). Based on those characteristics, two major therapeutic schemes have been applied clinically in cancer treatments: genotype-directed precision oncology, targeting specific genomic abnormalities of various types of cancer to provide individual treatment, and anti-tumor immunity, focusing on the components of tumor microenvironment, especially the immune system (15–19). Since potent metabolic regulatory properties of PPARs have made their modulators widely employed in the treatment of numerous diseases, including dyslipidemia, type 2 diabetes (T2D), and various metabolic disorders, scientists are now diving into their association with cancer (20). Compelling evidence is emerging on cancer cell proliferation and differentiation regulation by PPAR modulators, yet sometimes with contradictory results (21). Among all three subtypes, PPAR-γ has attracted relatively larger attention. For future reference of further studies on PPAR-γ’s cancer treating potential, this review focuses on the recent findings on the functions of PPAR-γ, current explorations and discoveries, as well as potential applications of PPAR-γ modulators in cancer field.

## 2 Functions of PPAR-γ

PPAR-γ, a subtype of PPARs, is most expressed in adipose tissue, with two isoforms PPARγ1 and PPARγ2, the former being more biologically and universally expressed in various tissues, while the latter is more restricted to adipose tissue, but could be induced by high-fat diet (HFD) in other tissues. This tight linkage between PPAR-γ and fat has significantly attributed to the multi-functions of PPAR-γ in metabolism, vascularization, inflammation, cell cycle regulation, differentiation, and migration, rendering it a special niche in the treatment of metabolic disorders and cancers.

### 2.1 Regulation of Lipid and Glucose Metabolism

PPAR-γ is highly implicated in metabolism of lipids, which is a potent facilitator of adipogenesis and fatty acid storage (10, 22). The activation of PPAR-γ decreases circulating lipids which are majorly triglycerides and free fatty acids. Meanwhile, it selectively promotes lipid uptake by inducing adipogenesis and increasing lipid storage (20). PPAR-γ is pivotal throughout the entire process of adipogenesis. It is an essential regulator of adipocyte differentiation, a probable participant in adipocyte self-renewal, and a requisite for mature adipocyte function (11). Alongside the metabolic changes of lipids induced by PPAR-activation, a series of adipokines is up-regulated. They participate in multiple actions, including insulin sensitivity regulation, inflammation, tumorigenesis, and global metabolism adjustment. Adiponectin may act on hepatic glucose output with additional vascular benefits on eNOS facilitated by other intermediates, e.g. shock protein 90 and Src homology region 2-containing protein tyrosine phosphatase 2, etc., through the PI3K-Akt pathway (20); resistin, leptin, and tumor necrosis factor-α (TNF-α), majorly functions in inflammatory reactions.

All mentioned adipokines, working individually or collectively, are factors affecting system insulin sensitivity. This collaborative effect on glucose metabolism is realized through the activation of genes responsible for insulin-dependent glucose uptake (GLUT4, IRS-1, IRS-2, and c-Cbl associated protein), and key genes involved in glucose-stimulated insulin secretion (GSIS), *via* the activation of PPAR-γ (10, 11, 20).

### 2.2 Inhibition of Inflammation and Tumorigenic Implications

Being a part of PPARs superfamily, PPAR-γ also actively regulates inflammation and immunity responses. On the genetic level, PPAR-γ may activate trans-repression on pro-inflammatory genes with SUMOylation. SUMOylation is a post-translational modification, namely the conjugation of PPAR-γ with Small Ubiquitin-like Modifier (SUMO) upon ligand activation. After a nuclear corepressor complex is bound, nuclear factor kappa B (NF-κB) target genes are trans-repressed, which stabilizes NF-κB in a repressed, promoter-bound state (10, 20). In addition, PPAR-γ may regulate the immune system through dendric cells (DC) and macrophages. It affects DC functions by altering antigen uptake, maturation, activation, migration, cytokine production, and lipid antigen presentation. In macrophages, PPAR-γ inhibits genes encoding pro-inflammatory molecules while activating the expression of anti-inflammatory mediators. It also manipulates cell differentiation to inhibit wild-type proinflammatory ‘M1’ macrophages, while facilitating the maturation of anti-inflammatory ‘M2’ macrophages, rendering anti-inflammatory effect bilaterally (11, 20).

Strong regulative properties of PPAR-γ on inflammation and immunity indicate its potential in cancer immunotherapy. Multiple signaling pathways may be engaged in anti-tumorigenic activities by the activation of PPAR-γ. In PPAR-γ-attenuated mice melanoma cells, the infiltration of the myeloid-derived suppressor cells (MDSCs) exhibits overall non‐specific inflammatory responses. A corrective effect is achieved upon the ligand-binding of PPAR-γ, and tumor growth is inhibited. This effect is achieved *via* the mTOR pathway, the subsequent blocking of MDSCs ROS overproduction, and possibly the RAGE pathway (23, 24). Macrophages are highly plastic and heterogeneous. In the context of cancer, tumor-associated macrophages (TAMs) are particularly abundant and pro-proliferative within tumors. They are engaged in immunosuppression and angiogenesis, supporting tumor growth and metastasis. When activated, according to Gionfriddo, G., et al., PPAR-γ can reduce the secretion of M1 pro-inflammatory and pro-tumor M2-cytokines without affecting macrophage polarization, yielding an anti-cancer effect (25). However, other studies contended the changes of polarization. Li, T., et al. found that in macrophages derived from human monocytic leukemia cell lines, a tumor suppressor called docking protein-1 (DOK1) can be activated with the activation of PPAR-γ. It was supposed to induce polarization of macrophages towards an inflammatory phenotype with increased release of pro-inflammatory cytokines and reduced PD-L1 expression (26).

### 2.3 Induction of Cell Differentiation

Differentiation grades are a critical criterium in assessing the malignancy of tumors, which holds great significance in prognosis and development of clinical treatment. PPAR-γ possesses the differentiation-inducing effect, which is associated with multiple different mechanisms yet has not been entirely elucidated (27, 28). The presence of PPAR-γ expression is required in tissue development, the placenta and heart, without which serious damage could be done. The deficiency of PPAR-γ, according to Barak et al., interferes with terminal differentiation of the trophoblast and placental vascularization, leading to severe myocardial thinning and death (29). In breast cancer cells, both *in vitro* and *in vivo*, the activation of PPAR-γ induces terminal differentiation of cancer cells into adipocytes and causes lipo-apoptosis with up-regulated expressions of C/EBPβ (30). Cells which go through epithelial-mesenchymal transition (EMT) and trans-differentiated afterward are growth-arrested, losing their cellular plasticity (31). PPAR-γ activation also causes degradation of β-catenin and inactivation of the downstream Wnt/β-catenin pathway, and thus increases ketogenesis by inducing mitochondrial 3-hydroxy-3-methylglutaryl-CoA synthase 2 (HMGCS2), a rate-limiting ketogenic enzyme in the synthesis of ketone body β-hydroxybutyrate (βHB), which contributes to the regulation of intestinal cell differentiation (32–34). As cancer cells are characterized by the symbolic energy metabolism reprogramming, namely the Warburg effect, ketogenesis contributes to intestinal cell differentiation *via* the inhibition of glycolysis, thus attenuating the progression of human colon cancer cells *in vitro (* 35). On the protein level, the activation of PPAR-γ promotes epithelial differentiation and stabilizes the differentiated phenotype by upregulating the expression of key proteins like keratins, E-cadherin, alkaline phosphatase, and developmentally-regulated- GTP-binding protein 1, carcinoembryonic antigen (CEA), differentiation-related gene-1 (DRG1), possibly through the interaction with Hic-5 which may serve as a PPARγ co-activator (10, 20).

### 2.4 Suppression of Cell Proliferation

Limitless cell proliferation with self-sufficient growth signals and insensitivity to contrary or anti-growth signals are the hallmarks of cancer tissue (36); thus, the inhibition of cell proliferation is a major constituent in the attenuation of tumor growth. The anti-proliferative effect from the activation of PPAR-γ is fulfilled by a variety of mechanisms, such as the induction of cell cycle arrest, the direct up-regulation of certain genes, and the activation of various signaling pathways involved in immunity and inflammatory responses.

Activated upon ligand-binding, according to Zurlo et al., PPAR-γ can interact with Sp1, stimulate p21(waf1/cip1) gene transcription, and consequently induce a G0/G1-phase arrest in human colorectal cancer and gastric carcinoma cells (32–34, 37). Multiple cyclins and cyclin-dependent kinases (CDKs) are positive regulators of cell cycle progression, and upon agonist-binding, PPAR-γ can facilitate cyclin D1 ablation, inducing cell arrest (38–40). Another study conducted on rats with breast cancer *in vivo* has showed the direct up-regulation of PTPRF gene expression by the activation of PPAR-γ, which in part inhibits tumor cell proliferation (41). Additionally, upon PPAR-γ activation, decreased levels of migration and invasion of human gastric adenocarcinoma cells *via* downregulating the ERK-signaling pathway (40) have also been demonstrated in a study through long-term patient investigation (42).

Multiple signaling pathways are implicated in the immuno-responses towards cancer cells induced by the activation of PPAR-γ: (1) the blockade of the RAGE signaling weakens the recruitment and accumulation of further myeloid cells, including MDSCs, and the inhibition on T and natural killer cells, ameliorating T cell tolerance and strengthening anti-tumor immunity (24, 43); (2) the inhibition of Toll-like receptor 4 (TLR4)-dependent mitogen-activated protein kinase (MAPK) pathway leads to the decreased activity of downstream NF-κB pathway (44); (3) the blocking of the mTOR signaling pathway not only downregulates proliferation, migration and invasion of mice prostate cancer and melanoma, but also upregulates apoptosis and autophagy both *in vitro* and *in vitro (* 23, 45), which were consistent with other studies confirming the increased production of reactive oxygen species (ROS) additively (23, 40, 46); (4) the co-activation and high expression of C/EBPα and PPAR-γ, with the former inhibiting Forkhead box C1 (FOXC1)’s promoter activity, an important cancer-associated gene in tumor, can significantly inhibit cell proliferation, migration, invasion, and colony information (47); (5) the activation of PPARγ-LXRα-ABCA1 pathway by lycopene has been demonstrated to inhibit both androgen-dependent prostate LNCaP cancer cells and androgen-independent prostate cancer cells DU145 and PC-3 (48).

### 2.5 Regulation of Cell Apoptosis

Another hallmark of cancer is the avoidance and evasion of programmed cell death. Canonical pathways of apoptosis include the intrinsic mitochondrial pathway and the extrinsic pathway mediated *via* the activation of cell surface death receptors. The intrinsic apoptotic signaling pathway primarily involves the activation of the proapoptotic Bcl-2 family members Bax and Bak, which facilitates the release of cytochrome c from the mitochondria and subsequent caspase-9 cleavage or activation, followed by the final cleavage or activation of the downstream effector caspases such as caspase-3 and -7, resulting in apoptosis. This is a pathway negatively regulated by several anti-apoptotic Bcl-2 family members, e.g., Bcl-2 and Bcl-XL. Apoptotic signaling through the extrinsic pathway is initiated by ligand-binding of death receptors which belong to the tumor necrosis factor (TNF) receptor superfamily, or the induction of trimerization of the said receptors. After that, the intracellular death domain of the death receptors subsequently recruits adapter proteins, forming a death-inducing signaling complex (DISC) which helps to recruit procaspase-8 to the DISC. When caspase-8 is activated, downstream effector caspases such as caspase-3 and - 7 will then take effect. The effector caspases can also be activated by death receptors indirectly through caspase-8-mediated cleavage of Bid, which facilitates Bax activation and the subsequent release of cytochrome c from the mitochondria. Thus, the Bid cleavage links both apoptotic pathways (40).

The apoptotic effect of PPAR-γ is exerted *via* both the intrinsic and extrinsic apoptotic pathways, either by downregulating the expression of anti-apoptotic BCL-2 genes, elevating pro-apoptotic factors Bax, and increasing endogenous POX expression, which is a redox enzyme localized in the mitochondrial inner membrane and mediates apoptosis through generation of reactive oxygen species (ROS) (49, 50); or increasing the activity of caspase-3, -8 and -9, *via* RAGE, mTOR, TLR4-dependent MAPK, PTEN-Akt and NF-κB signaling pathways (37, 43–45, 51); additively, there is the simultaneously increased expression of pro-apoptotic genes, such as growth arrest and DNA-damage inducible 153 (GADD153), c-Myc, metallothionein, and etc. (40).

### 2.6 Promotion of Tumorigenesis

Although flooding evidence supports the tumoricidal effect of PPAR-γ, its pro-tumorigenic potential cannot be ignored. Indeed, the counterpart’s testimony is also too grounded to be dismissed reasonably, given the consideration that most effects are achieved in a dose, concentration, cancer type and individual-specific manner. Current evidence on the pro-cancer effect of PPAR-γ revolves around the intercellular and extracellular adhesion, inflammation, metabolism, cell proliferation, rendering it a negative prognostic biomarker. The carcinoembryonic antigen (CEA) is an important component of the intercellular adhesive forces, and its over-expression can be pro-oncogenic, which may even induce the blockage of differentiation of rat myoblast cells. Upon ligand binding, PPAR-γ can induce an increase in CEA-dependent intercellular adhesion (10). In concert, the up-regulated expression of vascular endothelial growth factor A (VEGF-A) and Vimentin, a major intermediate filament protein that plays an important role in cell adhesion, migration, angiogenesis and neurite extension, is relate to the activation of PPAR-γ (52). Additionally, the inactivation of PPAR-γ by antagonists interferes with cancer cells’ adhesion to the extracellular matrix, disrupting survival signals, and thus inducing anoikis, a special form of apoptosis (40).

The activation of PPAR-γ displays a contradictory effect in immunotherapies equally. According to Wu, B., et al., the inactivation of PPAR-γ boosts the efficacy of αPD-L1 and αPD-1 antibodies in the treatment against murine mammary tumors (53). The activation of PPAR-γ in turn suppresses the expression and secretion of inflammatory factors, pro-inflammatory chemokines, which may reprogram the immune microenvironment of cancer to be less “inflamed”, consequently enabling resistance of cancer cells to immune-directed therapies (54).

Metabolic-wise, researchers have found that the activation of PPAR-γ could improve glucose and lipid metabolism, proving for the high demand of energy in the proliferation, migration and invasion of human prostate cancer cells and mice model (52). Interestingly, regarding factors involved in cell cycle, another study has found that the pro-apoptotic genes c-Myc and Bax are inhibited while cell-cycle regulators PCNA, cyclin D1 and COX-2 are promoted along with PPAR-γ activation (40, 55). In several studies, with the cell culture of human renal cell carcinoma, human prostate cancer xenografts in nude mice, as well as brain cancer *in vivo*, the increased expression of PPAR-γ are all prognostically negative and pro-metastatic (56–58).

**Figure 1:**
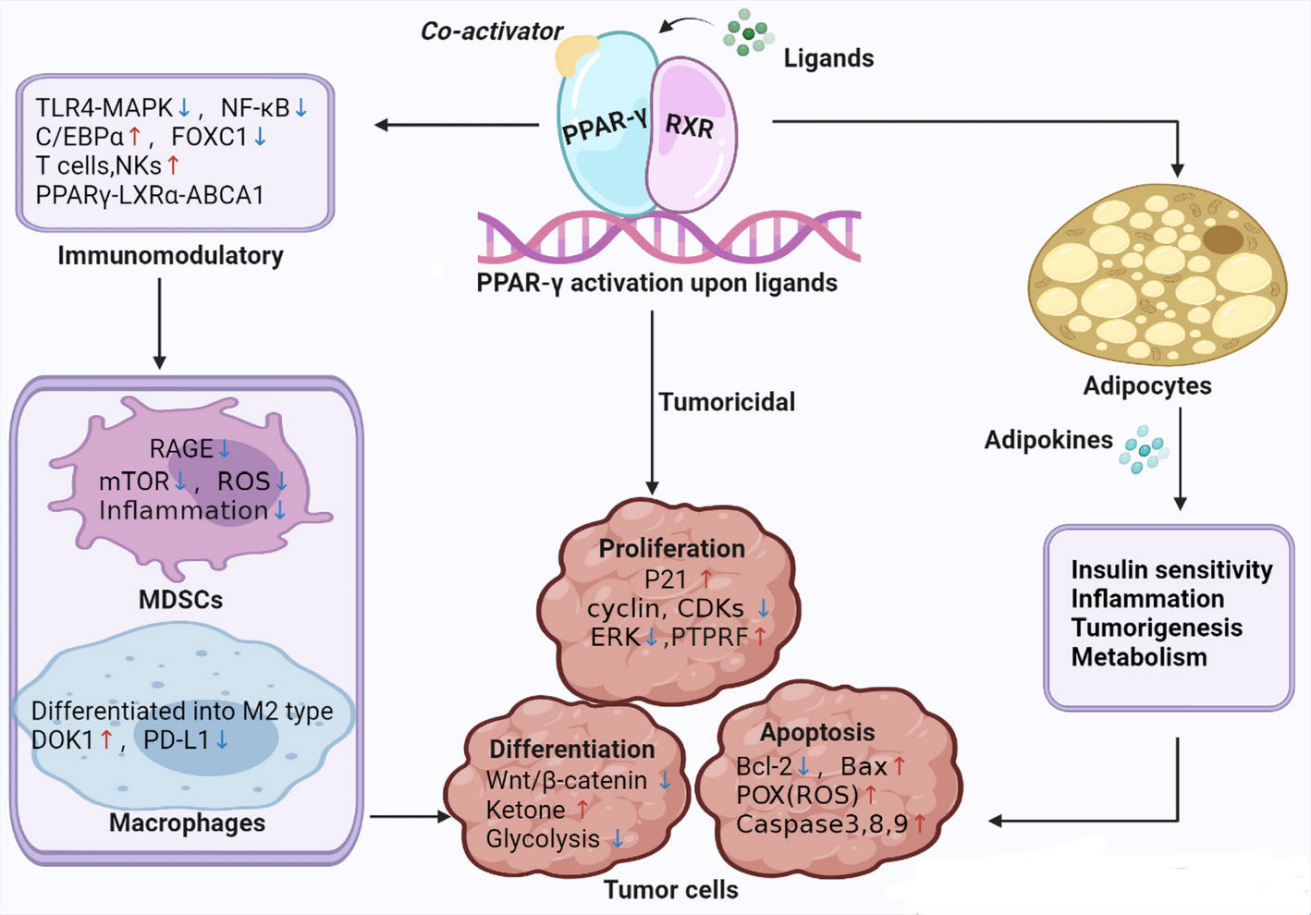
Main mechanisms of PPAR-γ in the regulation of tumor. TLR4-MAPK, Toll-like receptor 4-mitogen-activated protein kinases; mTOR, Mechanistic target of rapamycin; C/EBPβ, CCAAT/enhancer-binding protein beta; FOXC1, Forkhead box C1; LXRα, liver X receptor alpha; ABCA1, ATP binding cassette A1; NKs, Natural killer; CDKs, Cyclin dependent kinase; PTPRF, protein tyrosine phosphatase receptor type F; ERK, extracellular signal-regulated kinase; POX, Proline Oxidase; DOK1, downstream of tyrosine kinase; PD-L1, programmed death-ligand 1.

**Figure 1** illustrates the main mechanisms of PPAR-γ in the regulation of tumor.

## 3 Modulators of PPAR-γ

PPAR-γ is activated either endogenously or exogenously, respectively by polyunsaturated fatty acids, fatty acid derivatives such as 15-deoxy-delta-12,14-prostaglandin J2 (15d-PGJ2), nitrated fatty acids (10), and thiazolidinediones (TZDs) majorly, as well as other novel synthetic compounds with non-thiazolidinedione scaffold (27). PPAR-γ modulators have been used for multiple types of cancer, because it can inhibit tumor growth by directly interfering with related signaling pathways, or indirectly enhancing the tumor cell sensitivity to the radiotherapy, and get utilized in combination with other drugs to promote efficacy synergistically (**Table 1**).

**Table 1:** Summary of the PPAR-γ modulator in different types of cancer.

Modulator	Cancer type	Status	Main findings	Reference
**Ciglitazone**	Breast and Lung cancer	*In vitro*	Induced cyclin D1 ablation and cell cycle arrest.	(39, 59–61)
	Prostate cancer	*In vitro*	Ameliorated the aberrant intrinsic apoptotic activities.	(62)
	Prostate and Ovarian cancer	*In vitro*	Induced apoptosis.	(63)
	Prostate cancer	*In vitro*	Reduced prostate-specific antigen secretion.	(64, 65)
	Breast cancer	*In vitro*	Inhibited cyclin D1 and expression of estrogen receptor alpha.	(39, 60)
	Brain tumor	*In vitro*	Reduced tumor expansion and promoted differentiation and self-renewal.	(66)
**Troglitazone**	Breast cancer	*In vitro*	Inhibited NF-κB, activator protein-1 DNA binding, and matrix metalloproteinases.	(67)
	Prostate cancer	*In vitro*	Upregulated E-cadherin and glutathione peroxidase 3 levels.	(68)
	Colon cancer	*In vivo*	Modulated E-cadherin/beta-catenin system and promoted differentiation.	(68)
	Thyroid cancer	*In vitro*	Increased expression of sodium-iodide symporter and restore oradioiodine uptake.	(69–71)
	Pancreatic cancer	*In vivo*	Promoted mitochondria-mediated apoptosis and inhibited cell migration.	(72)
	Prostate cancer	*clinical phase II*	Decreased serum cancer-specific antigen level.	(73)
	Gastric and Breast cancer	*In vitro*	Increased cell apoptosis and inhibited growth.	(74, 75)
	Lung cancer	*In vitro*	Induced growth inhibition, in combination with cisplatin and paclitaxel.	(76, 77)
	Ovarian and Breast cancer	*In vitro*	Increased cell apoptosis.	(78–80)
	Colon and Oral squamous cancer	*In vivo*	Reduced aberrant formations.	(81, 82)
**Pioglitazone**	Glioma	*In vivo/vitro*	Decrease β-catenin level.	(83)
	Breast cancer	*In vivo/vitro*	Inhibit expressions of estrogen receptor and aromatase.	(83)
	Liver cancer	*In vitro*	Decreased RAGE level.	(84)
	Lung cancer	*In vivo/vitro*	Induced a metabolic switch: inhibited pyruvate oxidation and increased ROS levels. Induced cell cycle arrest.	(85)
	Colon cancer/Pancreatic cancer	*In vivo/vitro*	Suppressed angiogenesis.	(86, 87)
	NSCLC	*In vivo/vitro*	Inhibited NF-kB expression and angiogenetic activity.	(88)
	Liver cancer	*clinical phase I*	Prevented against radiation-induced cognitive decline.	(89)
	Melanoma	*Clinical phase II*	Stabilized remission.	(90, 91)
**Rosiglitazone**	Liver cancer	*In vitro*	Increased PTEN level and inhibited the PI3K/AKT pathway.	(92, 93)
	Melanoma and Breast cancer	*In vitro*	Inhibited the tumor growth.	(94, 95)
	Breast cancer	*In vivo/vitro*	Suppressed expression of NHE1.	(96)
	Breast cancer	*In vitro*	Induced apoptosis.	(97)
	Pancreatic cancer	*In vitro*	Induced apoptosis.	(98)
	Adrenocortical cancer	*In vitro*	Inhibited the proliferation by promoting autophagy.	(99)
	Prostate cancer	*In vitro*	Downregulated migration, invasion and PI3K/Akt activation.	(100)
	Colorectal cancer	*In vitro*	Reduced tumor migration and metastasis.	(101)
	Melanoma	*In vitro*	Suppressed Frizzled-1 (FZD1) and inhibited Wnt/β-catenin pathway.	(102)
	Pancreatic cancer	*In vivo*	Reversed the immune-suppressed state. Inhibited angiogenesis.	(103–105)
	Myeloma	*In vitro*	Inhibited angiogenesis.	(106)
	NSCLC	*In vitro*	Increased the expression of 15-PGDH.	(107)
	Liver cancer	*In vitro*	Downregulate Jab1 level.	(108)
	Liver cancer	*In vitro*	Enhanced TRAIL-induced apoptosis.	(109)
	NSCLC	*In vitro*	Suppressed mTOR signaling and activated tuberous sclerosis complex 2 (TSC2).	(110)
**Efatutazone**	Lung adenocarcinoma	*In vitro*	Facilitated the treatment.	(111, 112)
	Ductal carcinoma	*In vivo*	Induced a higher level of lactational and luminal cell differentiation and delayed cancer process.	(113)
**Balaglitazone**	Leukemia	*In vitro*	Elevated PTEN and reversed multidrug resistance.	(114)
**Resveratrol**	Uterine sarcoma	*In vitro*	Inhibit proliferation by blocking WNT/β-catenin pathway.	(115)
**Curcumin**	Diffuse large B cell lymphoma	*In vivo/vitro*	Induced cell apoptosis and G2 cell cycle arrest by inhibiting the Akt/mTOR pathway.	(116)
**Bavachinin (Bnn)**	Lung cancer	*In vitro*	Generated ROS and suppressed cancer growth.	(117)
**Cladosporols A/B**	Colon cancer	*In vitro*	Limited cancer cell proliferation.	(118)
**Alpha-Tocopherol**	Esophageal cancer	*In vivo*	Chemoprevention role by increasing PTEN and downregulating Akt.	(119)
**Hydroxysafflor Yellow**	Gastric cancer	*In vitro*	Promoted cell apoptosis.	(120)
**VSP-17**	Breast cancer	*In vivo/vitro*	Inhibited cancer metastasis.	(121)

Bcl-2, B-cell lymphoma-2; FLIP, FLICE-inhibitory protein; TRAIL, TNF-related apoptosis-inducing ligand; TNF, tumor necrosis factor; NF-κB, nuclear factor kappa B; JNK, c-Jun N-terminal kinase; ER, estrogen receptor; PTEN, phosphatase and tensin homolog deleted on chromosome ten; PGE2, Prostaglandin E2; BRCA1, Breast-Cancer susceptibility gene 1; RAGE, receptor for advanced glycation end products; PDK4, Pyruvate dehydrogenase kinase; ROS, reactive oxygen species; COX-2, cyclooxygenase; PI3K, phosphatidylinositol-3-kinase; ERK, extracellular signal-regulated kinase; NHE1, exchanger isoform-1; TIMP-1, Tissue inhibitor matrix metalloproteinase 1; MDR, multiple drug resistance; MDSC, myeloid-derived suppressor cells; HIF-1α, Hypoxia-inducible factor 1; EMT, epithelial-mesenchymal transition.

### 3.1 PPAR-γ Agonist: Thiazolidinediones Family

Thiazolidinediones (TZDs) are synthesized PPAR-γ agonists, also known as glitazones, which started drawing considerable attention since the discovery of the prototypical drug ciglitazone. Through PPAR-γ transactivation and trans-repression, by altering the expression of related genes to promote fatty acids storage in adipocytes, and consequently prioritizing the oxidation of carbohydrates, more specifically glucose, for cellular energy supply, TZDs are primarily employed in the treatment of type 2 diabetes (T2D) as insulin sensitizers. The first generation of TZDs includes troglitazone, which was invented and approved by FDA in 1997, only to be withdrawn from the market after a mere three years due to serious hepatotoxicity. Alarming side effects had led to the appearance of the second generation of TZDs (122). Derived from the parent compound thiazolidinedione, members of the second generation of TZDs, including pioglitazone and rosiglitazone, were developed by altering the metabolic product of ciglitazone, though the original one was never introduced into clinical use.

Recently, TZDs also present anti-cancer effects dependently or independently of PPAR-γ activation, both on the transcriptional and the protein level. Mechanisms are involved in cell cycle, apoptosis, hormonal reactions, and stromal regulation, including partial depletion of intracellular Ca2+ store, proteasomal degradation of related proteins to induce cell cycle arrest and apoptosis, transcriptional repression of sex hormone receptors, and decreased activation of macrophages (123, 124).

Before the elaboration on specific drugs, it is argued that there exist several lines of findings in the anti-tumor properties of TZDs in question of the dependence of PPAR-γ activation. First, the potency of effects exemplifies a manner irrespective of the activation levels of PPAR-γ, but more reliant on drug chemical structures, evidenced by the disparity of apoptosis-inducing ability between troglitazone and pioglitazone. Secondly, the susceptibility of tumor cells towards TZDs is in no strict accordance with PPAR-γ activation levels, where cancer with low expression of PPAR-γ respond better than those with over-expression. Thirdly, there is a considerable increase in the anti-tumor potency of the PPAR-γ-inactive analogues than their parent compounds, like Δ2TG and Δ2CG, which are developed from troglitazone and ciglitazone, respectively. Lastly, the apoptotic effect of TZDs and derivatives is unshaken by the siRNA-mediated knockdown of PPAR-γ in certain cell lines (123).

#### 3.1.1 Ciglitazone

Ciglitazone is the prototype of all TZDs developed in the early 1980s, which has never been approved for clinical application in diabetic treatments due to its weak therapeutic efficacy. However, this debutante led way of several potent insulin sensitizers, the analogues of which, troglitazone, pioglitazone and rosiglitazone, into the therapeutic market in the following years. Since the finding on the varied expression of PPAR-γ in cancer of different differentiation stages and its involvement in cell proliferation, however, ciglitazone has been striving to make a comeback into the medical field as a novel potential anti-tumor agent.

The anti-tumor effects exerted by ciglitazone extend into various perspectives, including cell cycle, apoptosis, and sex hormones, targeting different categories of cancers. Distinguished from its peers and derivatives, the effects of ciglitazone and its analogue links to PPAR-γ independent. During the crucial G1/S phase transition in cell cycle, ciglitazone can suppress cancer progression either by partially depleting intracellular calcium stores, consequently causing the deactivation of eukaryotic initiation factor 2 (eIF2) to inhibit translation initiation (125); or functioning as a cyclin D1-ablative agent *via* proteasomal degradation, which results in cell cycle arrest (39, 59–61). Furthermore, ciglitazone is also equipped with the capabilities of effecting apoptotic pathways both intrinsically and extrinsically. Through blocking the interactions between Bcl-2 and Bcl-xL with Bak and other pro-apoptotic proteins, ciglitazone can suppress the anti-apoptotic functions of Bcl-xL and Bcl-2, ameliorating the aberrant intrinsic apoptotic activities (62). TNF-related apoptosis inducing ligand (TRAIL) is a member of the tumor necrosis factor (TNF) family of cytokines, an extrinsic apoptosis inducer. By selectively inhibiting FLIP (FLICE inhibitory protein), an apoptosis-suppressing protein, ciglitazone can block early events in TRAIL/TNF family death receptor signaling and eliminate cancer cells with apoptosis *via* the extrinsic pathway (63).

Aside from the universal anti-proliferative effects concerning cell cycle and apoptosis, ciglitazone also possesses properties associated with sex hormone receptors. The impacts of hormone receptors on cancer status are distinctive of hormone-dependent cancers. In prostate cancer cells, ciglitazone and its analogue reduce prostate-specific antigen (PSA) secretion with and without a decrease in the expression of androgen receptor (AR), independent of PPAR-γ activation. However, in androgen-independent C4-2 prostate cancer cells, the ciglitazone-induced regulation of AR is PPAR-γ-dependent (64, 65). In breast cancer cells, alongside the aforementioned repressive effect on cyclin D1, the expression of estrogen receptor (ER) alpha is simultaneously inhibited by ciglitazone, possibly yielding a synergistic anti-tumor effect (39, 60).

Ciglitazone not only displays significant potential in monotherapy, but combines with other drugs to improve the efficacy. The combination of ciglitazone and RXR-α ligands promotes growth inhibition and apoptosis. Moreover, the co-administration of ciglitazone and drugs outside of conventional cancer medication, such as lovastatin and phenylbutyrate, triggers TNF-α-related apoptosis inducing ligand and gamma-radiation and the decrease of cancer cell viability (124).

Ciglitazone can also be a potential chemo-preventative agent due to its pro-differentiation effect. Multiple PPAR-γ agonists, including ciglitazone, are capable of reducing brain tumor stem cell expansion and promoting differentiation and self-renewal (66).

#### 3.1.2 Troglitazone

Troglitazone (TGZ), the first generation of TZDS, is a dual-agonist of PPAR-α and PPAR-γ, more strongly of the latter, which underlines its multi-functionalities in tackling inflammation on top of diabetes (126). It was the first-ever anti-diabetic drug with the mechanism of insulin sensitization, carrying the belief of reducing cardiovascular risks associated with diabetes upon addressing the primary metabolic defect (127, 128). However, after the linkage to the estimated 430 liver failures and the reported 63 liver failure deaths during the meagre span of three years since the FDA approval in 1997, TGZ was withdrawn from the market in 2000. And since then, extensive studies have been carried out on its hepatotoxicity, the understanding from which in turn took a twist and shed a light on the treatment of cancer by TGZ’s anti-tumor properties (129).

On the transcriptional level, TGZ exerts effects with transactivation and trans-repression of genes, with the former majorly mediating anti-proliferation and -migration, and pro-differentiation, and the latter involving in inflammatory responses (124). In MCF-7 breast cancer cell line, TGZ reverses the effects induced by 12-O-tetradecanoylphorbol-13-acetate (TPA), by inhibiting nuclear factor κB (NF-κB) and activator protein-1 DNA binding, and inhibiting matrix metalloproteinases (MMPs) which play a significant role in tissue remodeling, through a PPAR-γ-dependent mechanism (67). Additionally, in prostate cancer PC-3 cell line, cancer growth is attenuated *via* the up-regulation of E-cadherin and glutathione peroxidase 3 (GPx3) upon TGZ administration, in a dose- and PPAR-γ-dependent manner, with the downregulation of which being characteristic of epithelial-mesenchymal transition (EMT) (68). Similar results are shown in another study conducted on xenograft mice with human colon cancer cells, where both the E-cadherin/beta-catenin system and the Drg-1 gene expression were modulated, yielding a differentiation-promoting effect (130). Among all cancer, Differentiated Thyroid Carcinoma (DTC) holds a special place for TZDs, because follicular thyroid cancer is the only known neoplasm associated with a PPAR-γ fusion gene product with over 30% follicular thyroid carcinoma expressing PAX8/PPAR-γ (124). Aside from the traditional anti-tumor properties of anti-proliferative, proapoptotic, and differentiating effects, the increased expression of sodium-iodide symporter and restoration of radioiodine uptake renders TGZ a potential agent in treatments against DTC, especially those resistant to conventional therapies (69–71).

Whereas anti-tumor evidence has emerged outside the PPAR activating property of TGZ where no ligand-binding of PPAR is detected. By TGZ’s direct targeting epidermal growth factor receptor (EGFR) to induce its internalization and degradation by the endo-lysosomal degradation machinery, growth arrest of tumor cells can be achieved by the subsequent inhibition of EGF-induced Akt phosphorylation (124, 131). In another study on pancreatic cancer *in vivo*, TGZ promotes mitochondria-mediated apoptosis and moderately inhibits cell migration *via* the JNK pathway without marked adverse effects, in a PPAR-γ-independent manner (72).

Furthermore, there is a mixed result in phase 2 trials in demonstration of the clinical efficacy of TGZ in monotherapy. Little to no improvement is witnessed in studies on metastatic colon cancer and breast cancer, while a significant decrease in serum cancer-specific antigen is detected in prostate cancer patients administrated with TGZ (73, 132, 133).

Despite the currently discouraging data from TGZ monotherapy, the results from the co-administration of troglitazone with other compounds are preferable. Evidence has been emerging in diverse categories of medication: the joint use of RXR-α ligands and TGZ displays apoptotic and growth inhibitory effect in gastric carcinoma and breast cancer cell lines (74, 75); chemotherapeutic drugs, including cisplatin and paclitaxel, can induce growth inhibition of lung cancer cell lines, in synergy with TGZ (76, 77); in ovarian and breast cancer cell lines refractory to conventional therapies, combined administration of cell signaling molecules, including TRAIL (TNF related apoptosis inducing ligand), and TGZ achieved promising apoptotic results synergistically (78–80); among other studies, various medication, including statin (lovastatin), NSAIDs (aspirin), estrogen modulator (tamoxifen) and x-ray, have all been reported to work synergistically with TGZ against thyroid cancer, glioblastoma cancer, lung cancer, breast cancer and cervix cancer as anti-tumor agents (134–138).

Chemo-preventative effect of TGZ against cancer has also been contended. In chemically induced rat colon and oral squamous cancer model, TGZ administration significantly reduces aberrant formations and activities (81, 82).

#### 3.1.3 Pioglitazone

Pioglitazone (PGZ), derived from ciglitazone as a second-generation TZD, entered the US market for therapeutic use for type 2 diabetes in 1999, and shows therapeutic value in monotherapy and in co-administration as an adjuvant agent to conventional medication against various categories of cancer.

Several studies have supported that when administered alone, PGZ shows certain anti-tumor therapeutic effects both dependent on its PPAR-γ activation property and independent. In a PPAR-γ-dependent manner, PGZ decreases the expression level of β-catenin protein, a crucial carcinogenic mediator, and suppresses glioma cells growth and invasion both *in vitro* and *in vivo* as in mice xenograft (83). In breast cancer, the increased level of aromatase has been associated with heightened inflammation and a worse prognosis. The activation of PPAR-γ by PGZ can halt the progress of breast cancer *via* multiple mechanisms, targeting estrogen receptor (ER) and aromatase, either by inhibiting ER expression through the PTEN pathway or inducing proteasome-dependent degradation of ER, or inhibiting aromatase *via* the PGE2 and BRCA1 pathways (139). The elevated expression of receptor for advanced end glycation products (RAGE) has been reported in the pathological biopsy of human HCC tissues. After being treated with PGZ, there is the increased expression of PPAR-γ and decreased expression of RAGE of HCC cells in a dose-dependent manner, which indicates an association between PGZ administration and the inhibition on the growth and invasion of HCC cells (84). The significant metabolic regulating property of PPAR-γ can also contribute to the anti-tumor effect. According to Srivastava et al., the activation of PPAR-γ by PGZ induces a metabolic switch, inhibits pyruvate oxidation, by suppressing pyruvate dehydrogenase kinase 4 (PDK4) or β-oxidation of fatty acids, causes a marked increase in reactive oxygen species (ROS) levels, and eventually cell cycle arrest in lung cancer cells (85). Independent of PPAR-γ activation, there is evidence indicating the suppression of the expression of cyclooxygenase-2 (COX-2) and interleukin-8 (IL-8) after the administration of PGZ, exemplifying an anti-angiogenic effect on pancreatic cancer cells *in vitro* and colon cancer cells *in vivo (* 86, 87). Another *in vitro* study also revealed an interesting anti-tumor effect of PGZ independent of PPAR-γ activation. According to Saiki et al., the inhibitory effect of PGZ on the growth of human leukemia cell lines shows a selective pattern in leukemic, rather than normal human hematopoietic progenitor cells (140). Another study also on leukemia has revealed further evidence on PGZ’s anti-cancer effect in combined treatment with arsenic trioxide (ATO), which may be achieved *via* the suppression of PI3K/Akt pathway (141). Meanwhile, a group of Japanese scientists found that used alongside Cisplatinum (CDDP), PGZ could arrest human osteosarcoma cell’s chemoresistance, prohibiting cell proliferation and controlling necrosis (142). As for studies conducted *in vivo*, PGZ has also yielded promising performance in suppressing the progress of various cancer. In addition to anti-proliferative and anti-metastatic effect of PGZ on the aforementioned mice xenograft of human pancreatic cancer and colon cancer, there is also evidence of its anti-angiogenetic efficacy targeting non-small cell lung cancer (NSCLC) *via* PPAR-γ activation and the subsequent inhibition of NF-kB transcriptional activity (88). Similar to its analogues, PGZ also shows a chemo-preventative asset: neoplasm occurrence is suppressed in chemical-induced early-stage HCC when dosed with PGZ (143). This protective effect is also corroborated by a phase I clinical trial, where researchers found PGZ is well tolerated in prevention against radiation-induced cognitive decline (RICD) among patients undergoing radiotherapy (89).

Several phase II clinical trials were set out to test the efficacy of PGZ as an adjuvant agent in treating patients of refractory or advanced cancer. A combinational scheme consisting of trofosfamide, rofecoxib (a COX-2 inhibitor currently not in market) and clinically relevant doses of PGZ has exerted encouraging results in patients with chemo-refractory melanoma and soft tissue sarcoma, as well as advanced vascular malignancies, bringing stabilization and remission (90, 91).

Despite all the positive evidence, unfortunately, clinical data are depicting another not-so-inspiring picture. Dosage of PGZ in T2D patients has been suggested to be associated with risk of bladder cancer (144–146), and the longer of the term, the higher of the risks (147, 148). Yet further studies shall be carried out on the specific histological types of neoplasms, as well as the differences and biases between dosage course, diabetic pathology, as evidenced by others contending the decreased incidence of breast cancer (149), the unaffected risks of bladder cancer with short-term dosage (150), and no incidence of malignancy in normal urothelial transitional epithelium (NUTE) cells treated with PGZ (151). Concerns on the pro-tumorigenic effect PGZ has been witnessed to express offer another explanation: the dichotomized behavior, where the activation of PPAR-γ inhibits the invasion of cancer cells yet fuels the tumorigenic transition of the myeloid lineage (152). The “permissive” cell condition theory brought forward by Annicotte et al., where PGZ only effect as an anti-tumorigenic agent in the presence of valproic acid, an HDAC inhibitor, can counteract the mentioned pro-tumorigenic effects (153).

#### 3.1.4 Rosiglitazone

Rosiglitazone (ROSI) is another high-affinity PPAR-γ agonist, a second-generation TZD, originally developed as an insulin sensitizer for the treatment of diabetes. ROSI was patented in 1987 and approved for medical use in 1999, however, following its annual sales peak in 2006 at approximately $2.5-billion, it diverged onto a slope of falling profits as sales plummeted to just $9.5-million in 2012 after a 2007 meta-analysis linking the use of ROSI as an anti-diabetic with increased risks of cardiovascular incidents and heart attacks (154). Some urged FDA to take ROSI off the market, and from 2011 to 2013, stricter restrictions were forced by FDA on its purchase; but later in 2013, those restrictions were lifted after FDA’s review on a 2009 trial failing to show the alleged heart attack risks.

The anti-cancer effect upon PPAR-γ activation made researchers interested in this property of ROSI, and recent data has been encouraging. Marked results are being observed on various human malignant tumors, including gastric, breast, colorectal, adrenocortical and pancreatic cancer types; *in vivo*, ROSI also exhibits a preventative effect against multiple cancers (155). Like other members of the TZDs family, ROSI’s anti-cancer effect is yielded *via* both PPAR-γ-dependent and PPAR-γ-independent pathways, as well as both in monotherapy and in combined treatment with chemotherapeutics. In a PPAR-γ-dependent manner, the anti-tumor activities of ROSI include the induction of apoptosis and autophagy, proliferation and metastasis inhibition, amelioration of multidrug resistance (MDR), lifting of immune suppression, and inhibition of angiogenesis.

##### 3.1.4.1 PPAR-γ-Dependent Anti-Cancer Effects

The regulation of cell proliferation relies heavily on diverse signaling pathways, among which there are two important ones, the PI3K/AKT/mTOR and the MAPK/ERK pathways. There is a binding domain within the PTEN promoter, which is a natural inhibitor of the PI3K/AKT pathway, and ROSI increases the expression of PTEN through activating PPAR-γ in HCC cell lines (92, 93). As for the MAPK/ERK pathway, fulfilling the communication of signals from a membrane receptor to the cell nucleus, activated PPAR-γ halts the phosphorylation of ERK, thus inhibiting the tumor growth (94, 95). Apoptotic cell factors are vital participants in the process of cell apoptosis, hence the inhibition of proliferation, and evidence is accumulating on the effects of ROSI in this perspective. Among a plethora of factors, there is FasL, inducing apoptosis by binding to its receptor Fas, whose expression ROSI is observed to enhance (156); COX-2’s overexpression may be pro-tumorigenic, and its inhibition has been demonstrated to improve therapy outcome in colon cancer, upon the activation of PPAR-γ by ROSI, and vice versa (93, 98); NHE1, a osmotic homeostatic regulator whose decreased expression give rise to tumor cell death sensitization, whose promoter has a PPRE that PPAR-γ can bind to and inhibit its expression, has been reported to be significantly decreased in expression by ROSI in T2DM patients with breast cancer (96); BRCA1, a tumor suppressor gene whose overexpression induces apoptosis, is heavily PPAR-γ-responsive, whose protein expression can be promoted to induce apoptosis by ROSI administration in human MCF-7 cells (97); the Bcl-2 family is a potent modulator of cell apoptosis with both inhibiting and accelerating gene products, and ROSI induces apoptosis by upregulate the expression of one accelerating factor, Bax (98). Tumor cell apoptosis could be counteracted by autophagy, where apoptotic mediators get degraded; on the other hand, excessively activated autophagy causes cell death (157, 158). In an *in vitro* study, ROSI inhibits the proliferation of adrenocortical cancer cells by promoting autophagy through increasing the expressions of beclin-1 and Lamp-1 (99).

Metastasis is a distinctive behavior of malignant tumors, and a major reason for failed treatments. According to a recent study, ROSI is capable of downregulating C-X-C motif chemokine 12 (CXCL12)-induced migration, invasion and PI3K/Akt activation in a prostate cancer cell line, through the inhibition of the CXCL12/C-X-C chemokine receptor type 4 (CXCR4) axis (100). Apart from CXCR, ROSI has been found to suppress the expression of some migration-associated genes, including MMP-7, COX-2 and TIMP-1, consequently reducing tumor metastasis (101).

Multidrug resistance (MDR) is another key issue in the way toward satisfying treatment results. Encouragingly, ROSI exerts reversing effects in chemo-resistant ovarian cancer and melanoma cells *via* suppression of Frizzled-1 (FZD1), which enhances the inhibition of Wnt/β-catenin pathway, and the subsequent decreased expression of MDR1/P-gp, which is a common cause of MDR (102).

PPAR-γ has been known for its anti-inflammatory properties, majorly contributed by the reduction of inflammatory cytokines; in a tumor microenvironment, tumor-associated inflammation could result in immune suppression involving T regulatory cells and myeloid-derived suppressor cells (MDSC), whose accumulation is a great threat to a successful cancer treatment. ROSI can reverse the immune-suppressed state by limiting early MDSC accumulation and intra-tumoral T regulatory cells in pancreatic cancer mice model (103, 104).

Angiogenesis is a vital process in tumor growth and metastasis. Basic fibroblast growth factor (bFGF) and VEGF, two mitogenic cytokines, are highly responsible for endothelial cell growth and differentiation, hence angiogenesis. In a non-tumor model, ROSI has been reported to inhibit the activities of those two factors; in myeloma cell line, ROSI inhibits insulin-like growth factor 1 (IGF-1) or HIF-1α, which could boost VEGF’s pro-angiogenesis effects, *via* PI3K/AKT and ERK signaling in a PPAR-γ-dependent fashion (105, 106).

##### 3.1.4.2 PPAR-γ-Independent Anti-Cancer Effects

Despite its nature being a PPAR-γ agonist, ROSI could still partially exert its anti-tumor effects even with PPAR-γ inhibitor or siRNA knockdown.

Among all effects, induction of apoptosis may be the most preserved without activating PPAR-γ pathway, which could be achieved *via* inhibition of nuclear factor kappa-light-chain-enhancer of activated B cells (NF-κB) pathway and prostaglandin E2 (PGE2), as well as regulation of other apoptosis-associated cell factors. HCaRG, a novel calcium-regulated gene, is found to be in control of cell proliferation and differentiation, and has been reported to under-express in cancer cells and more expressed in normal tissue adjacent in patients with renal cell carcinomas (159). According to Solban et al., ROSI induces apoptosis by inhibiting NF-κB activity through upregulating HCaRG (160). 15-PGDH is a tumor suppressor gene which is responsible for inactivating PGE2, and limited degradation of the latter might lead to increased tumor growth not only by promoting apoptosis resistance, but also angiogenesis, tumor invasiveness, as well as inhibiting immune surveillance. Hazra et al. indicated that ROSI increases the expression of 15-PGDH to inhibit NSCLC growth (107). Other cell factors in the play of apoptosis include Jab1, a potential oncogene and critical in tumorigenesis, which could be down-regulated by ROSI in liver cancer cells (108); death receptor 5 (DR5), a member of the tumor necrosis factor (TNF)-receptor superfamily, which can be activated by TNF-related apoptosis inducing ligand (TNFSF10/TRAIL/APO-2L), and transduces apoptosis signal, and to be found up-regulated by ROSI to enhance TRAIL-induced apoptosis in various cancer cells (109); tuberous sclerosis complex 2 (TSC2), a tumor suppressor protein and modulator of the mTOR pathway, could be activated by ROSI with the consequent suppression of mTOR signaling, cell cycle halting, and inhibits growth of NSCLC cells (110).

Epithelial-mesenchymal transition (EMT) is a process highly associated with cancer progression and metastasis, where epithelial cells gain migratory and invasive properties (161). MET is the opposite. According to Tai et al., ROSI inhibits tumor proliferation and metastasis *via* inducing MKP-1 through promoting MET, consequently suppressing both tumor proliferation and metastasis (162).

##### 3.1.4.3 ROSI in Monotherapy and Combined Treatments

There are a plethora of studies focusing on utilizing ROSI in monotherapy as well as an adjuvant drug in combined administration with other treatments, which is showing a promising picture when put together. In cell cultures and xenografts, ROSI exhibits anti-tumor functions, involving apoptosis, cell growth, differentiation, migration, invasion, and tumor associated angiogenesis inhibition, as well as immune system regulation, in various malignancy, and some of them have entered phase II trials. When used in combination, ROSI exerts an enhancing effect upon chemo drugs or radiosensitivity, and/or a drug-resistance rescuing function. Several phase I cancer clinical trials are carrying out ROSI’s effects associated with chemotherapeutics (155).

#### 3.1.5 Other Modulators of the TZDs Family

Apart from the aforementioned well-known members of the TZDs family, other modulators in this category have been reported to own certain anti-tumor properties, including the amelioration of multidrug resistance, differentiation induction, etc.

Efatutazone is a novel PPAR-γ agonist and of the third generation of TZDs. Two separate studies by Ni et al. both reported that Efatutazone facilities the treatment of EGFR-TKI-resistant lung adenocarcinoma, by promoting the protein expression of PPAR-γ and phosphatase and tensin homolog (PTEN), causing the inactivation of the Akt pathway without affecting the transcriptional levels, which exerts a synergistic effect with LXRα, a member of another class of nuclear hormonal receptor reported being potential targets for the prevention and treatment of multiple cancers, agonist T0901317 (111, 112). Ductal carcinoma *in situ* (DCIS), a pre-invasive breast lesion, is the precursor of invasive ductal carcinoma. Efatutazone with short-term and low-dose administration in MCFDCIS xenograft and C3(1)/Tag transgenic mice induce a higher level of lactational and luminal cell differentiation and delay this process, reportedly to having activated PPAR-γ and subsequently downregulating Akt phosphorylation but leaving the ERK pathway unaltered (113). Balaglitazone is another member of the TZDs family. Upon treatment with balaglitazone, the expression of PPAR-γ and PTEN elevated, which leads to the P-glycoprotein-mediated multidrug resistance partly reverses in doxorubicin-resistant human myelogenous leukemia (K562/DOX) cells (114).

### 3.2 Non-TZD PPAR-γ Modulators

Except for TZDs, there are other PPAR-γ ligands uncovered with anti-cancer properties.

The overactivation of WNT/β-catenin pathway has been associated with oxidative stress and inflammation, consequently contributing to carcinogenesis. It has been suggested that in gliomas, canonical WNT/β-catenin pathway and PPAR-γ act in an opposed fashion (163). Resveratrol, a polyphenolic phytoalexin extracted from grape, inhibits uterine sarcoma cells proliferation by blocking WNT/β-catenin pathway in a PPAR-γ-dependent manner (115). Another PPAR-γ agonist, Curcumin, can inhibit the WNT/β-catenin pathway, as well as control circadian clocks relevant to this particular pathway, to restrict the growth of tumor (164). In addition, in diffuse large B cell lymphoma, Curcumin could also induce cell apoptosis and G2 cell cycle arrest by inhibiting the Akt/mTOR pathway (116). Bavachinin (Bnn) is extracted from a type of Chinese herb, named Psoraleacorylifolia. Bnn promotes the activation of PPAR-γ and generates ROS to suppress the growth of A549 lung cancer cell line (117). Cladosporols A is a natural metabolite of Cladosporium tenuissimum, which can fully activate PPAR-γ, similar to Rosiglitazone, and Cladosporols B is the oxidated product of the former, which owns partial PPAR-γ agonizing effect. Both of them exemplify the capacity to limit the proliferation of colon HT-29 cancer cells while the latter is more efficient (118). Hu et al. demonstrated that Alpha-Tocopherol, one component of Vitamin E, targets PPAR-γ and increases the expression of its downstream PTEN whereas downregulates Akt in rat esophageal cancer model induced by N-nitrosomethylbenzylamine (NMBA), indicating the chemoprevention role of Alpha-Tocopherol in early stage of esophageal cancer (119). Hydroxysafflor-Yellow is another PPAR-γ ligand originated from safflower. It is effective in limiting gastric cancer cells BGC-823 proliferation through apoptosis mechanism (120). Triple-negative breast cancer (TNBC) is an aggressive subtype with the absence of estrogen receptor (ER), progesterone receptor (PR), and human epidermal growth factor receptor-2 (HER2). Despite the declining mortality of breast cancer globally, owning to earlier diagnosis, TNBC subtype still causes high fatality, with metastasis and relapse being major clinical features in the late stage of TNBC and causing death directly. A novel PPAR-γ agonist VSP-17 targets EMT, indicated by elevated E-cadherin level, successfully inhibiting the metastasis of MDA-MB-231 cell line and xenograft model (121).

## 4 Discussion and Conclusion

Cancer has long been regarded as the most detrimental disease for its high morbidity and mortality. However, it is gratifying that many novel therapeutic targets, such as PPAR-γ, have been proved to show great potential in the inhibition of tumor proliferation and metastasis. By analyzing recent basic research, we have elucidated the functions of PPAR-γ to interpret the underlying mechanisms of PPAR-γ modulators in exerting anti-tumor effect. PPAR-γ regulates metabolism of lipid and glucose, inhibits inflammation and immunity responses, suppresses cell proliferation and induces cell differentiation and apoptosis. It is reported that upon PPAR-γ activation, the fatty acid oxidation in T cells are increased, and the subsequent increase in the proliferation of Tumor-Reactive CD8 + T Cells facilitates the efficacy of anti-PD-1 therapy (165). On the contrary, PPAR-γ appears to promote tumorigenesis *via* enhancing intercellular adhesion and restraining apoptosis. With the expression and secretion of inflammatory factors and pro-inflammatory chemokines suppressed, resistance to immunotherapy is likely to form in a PPAR-γ dependent manner, which immensely diminishes anti-tumor potential of wild type T cells compared with PPAR-γ-/- T cells (166). Moreover, PPAR-γ modulators can be divided into TZDs family which comprises three generations and non-TZD natural compounds mainly derived from herbs and plants. Based on the analysis of laboratory results and clinical study, we can conclude that PPAR-γ modulators play an auxiliary role rather than a leading role in tumor treatment. Compared with promising but paradoxical performance in weakening cancer proliferation and metastasis, the ability of PPAR-γ modulators to reinforce the tumor cell sensitivity to the therapies including radiotherapy, chemotherapy and TKIs is more pragmatic and closer to clinical application. In addition, whether the anti-tumor mechanism of TZDs is realized *via* PPAR-γ and which tumors are prone to PPAR-γ modulators remain to be further discovered. By establishing this thorough review on PPAR-γ modulators, we hope to provide valuable insight into how we can better tackle cancer in the future.

## Author Contributions

Conceptualization, ZL and PW. Resources, XW and PW. Writing—original draft preparation, TC, MW and KY. Writing—review and editing, TC, MW, XW, KY, and ZL. Supervision, ZL and PW. Project administration, PW. Funding acquisition, PW. All authors contributed to the article and approved the submitted version.

## Funding

This work was funded by Young Scientist Program by Beijing University of Chinese Medicine (Grant No. BUCM-2019-QNKXJ-C014), the Double First Class Construction Funds of Discipline of Integrated Traditional Chinese and Western Medicine of Beijing University of Chinese Medicine, and the Xin’ao Award Fund of Beijing University of Chinese Medicine (2019-XAJLJJ-006).

## Conflict of Interest

The authors declare that the research was conducted in the absence of any commercial or financial relationships that could be construed as a potential conflict of interest.

## Publisher’s Note

All claims expressed in this article are solely those of the authors and do not necessarily represent those of their affiliated organizations, or those of the publisher, the editors and the reviewers. Any product that may be evaluated in this article, or claim that may be made by its manufacturer, is not guaranteed or endorsed by the publisher.

## Glossary

**Table:**

5-LO	5-lipoxygenase
ACO	acyl-CoA oxidase
ACS	acyl-CoA synthetase
Akt	protein kinase B
AMP	adenosine monophosphate
AMPK	AMP-activated protein kinase
AP-1	activator protein-1
APOA1	apolipoprotein A1
APOA2	apolipoprotein A2
APOA5	apolipoprotein A5
APOC3	apolipoprotein C3
Bcl2	B-cell lymphoma-2
CACs	cancer anorexia cachexia syndromes
CKD4	cyclin-dependent kinases4
CLL	chronic lymphocytic leukemia
Cox	cyclooxygenase
CPT1	carnitine palmitoyltransferase 1
CPT2	carnitine palmitoyltransferase 2
CSC	cancer stem cell
CTL	cytotoxic T lymphocyte
CYP2C	cytochrome P450 2C
CYP1B1	cytochrome P450 1B1
DNMT1	DNA (cytosine-5-)-methyltransferase-1
EETs	epoxyeicosatetrienoic acids
eNOS	endothelial nitric oxide synthase
ER stress	endoplasmic reticulum stress
ERK	extracellular regulated protein kinases
FABP1	fatty acid-binding protein 1
FABP3	fatty acid-binding protein 3
FA	fatty acid
FAO	fatty acid oxidation
FLAP	5-lipoxygenase activating protein
FoxO	forkhead box O
FSAN	fatty acid synthes
GSK3	glycogen synthase kinase3
HEI-OC1	house ear institute organ of corti1
HIF1α	hypoxia inducible factor-1α
HDL	high-density lipoprotein
HMGCS2	hydroxymethylglutaryl CoA synthase 2
HNPGL	head and neck paraganglioma
IFNγ	interferon gamma
IκB	inhibitor of NF-κB
JNK	c-Jun N-terminal kinase
MAPK	mitogen-activated protein kinase
MMPs	matrix metalloproteinases
mTOR	mammalian target of rapamycin
MSC	mesenchymal stem cells
MVA	mevalonate
MYC	avian myelocytomatosis virus oncogene homolog
MyD-88	myeloid differentiation factor 88
NADPH	nicotinamide adenine dinucleotide phosphate
NF-κB	nuclear factor κB
NLRP3	nucleotide-binding domain, leucine-rich-containing family, pyrin domain-containing-3
NOX	NADPH oxidase
NSCLC-induced-CACs	nonsmall-cell lung cancer-induced-cachexia syndrome
OXPHOS	oxidative phosphorylation
PI3k	phosphatidylinositol 3-kinase
PKM2	Pyruvate kinase isozyme type M2
PRMT6	protein arginine N-methyltransferase
PD-1	programmed cell death protein 1
PLTP	phospholipid transfer protein
PPAR	peroxisome proliferator-activated receptors
ROS	reactive oxygen species
RXR	retinoid X receptor
SREBP1c	sterol regulatory element-binding transcription factor 1 c
STAT3	signal transducer and activator of transcription 3
TLR-4	Toll-like receptor-4
TRAIL	tumor necrosis factor-related apoptosis-inducing ligand
VEGF	vascular endothelial growth factor
